# A brief mindfulness‐ and compassion‐based parenting programme delivered via instant messaging: Results and implications from two randomised controlled trials on reducing parental stress

**DOI:** 10.1111/aphw.70094

**Published:** 2025-11-29

**Authors:** Doris L. W. Lam, Winnie W. S. Mak, Ben C. L. Yu

**Affiliations:** ^1^ Department of Psychology The Chinese University of Hong Kong Shatin Hong Kong; ^2^ Department of Applied Social Sciences The Hong Kong Polytechnic University Hung Hom Kowloon Hong Kong

**Keywords:** compassion, instant text messaging, mindfulness, parenting stress

## Abstract

High levels of parental stress can adversely affect both parents and children, leading to negative psychological outcomes. The present study consists of two randomised controlled trials investigating the effectiveness of a 14‐day mindfulness‐ and compassion‐based parenting programme delivered through instant text messaging in reducing parenting stress. Studies 1 and 2 recruited 222 and 271 parents in August 2021 and January 2022, respectively. All participants had at least one child studying in nursery or primary school. Participants were randomly assigned to either an intervention group or a waitlist control group. Assessments were conducted before the intervention, immediately following the intervention and 14 days after the intervention. During the 14‐day intervention, 14 audio recordings (ranging in length from 12 to 17 min) introducing parenting skills as well as mindfulness and compassion exercises were sent to the participants through WhatsApp and Signal. The primary outcome of both studies was parenting stress. Mental well‐being, interpersonal mindfulness in parenting and parenting behaviours were measured as secondary outcomes. Data were analysed using linear mixed models with full maximum likelihood estimation. A significant time by group interaction effect on parenting stress was found in both studies. In both studies, intervention effects on parenting stress were not sustained at 14‐day follow‐up. The results of the present study provided evidence that a brief, instant messaging‐based parenting programme has the potential to reduce parenting stress.

## INTRODUCTION

Parenting is a rewarding yet challenging experience that often comes with significant stress. High levels of stress can negatively impact parenting practices, leading to harsher disciplinary methods and less emotional support for children, which can create a cycle of stress and adverse psychological outcomes for both parents and children (Deater‐Deckard, 1998; Dumas, 2005). Identifying effective interventions that can alleviate parental stress and improve family well‐being is crucial.

Mindfulness‐based interventions (MBIs) have emerged as a promising approach for enhancing mental health (Kabat‐Zinn, 2003; Spijkerman et al., 2016). Mindfulness‐based practices involve cultivating one's present‐moment awareness by paying attention, on purpose, in the present moment, nonjudgmentally (Kabat‐Zinn, 2003). In recent years, MBIs have increasingly integrated principles from implementation science to enhance their accessibility and effectiveness (Dodge et al., 2024). Implementation science aims at understanding and improving the processes that facilitate the adoption and integration of evidence‐based practices in the community. This framework has been instrumental in guiding the development and dissemination of MBIs. Adopting innovative digital delivery systems to improve the mental health of various populations addresses the growing need for flexible, scalable solutions in mental health care.

Meta‐analyses found support for the effectiveness of online MBI in reducing various psychological symptoms of stress, anxiety and depression as well as enhancing mental well‐being in both clinical and nonclinical samples (Compen et al., 2018; Jayawardene et al., 2017; Spijkerman et al., 2016); and these gains remained stable at follow‐up (Krusche et al., 2012). The effectiveness of online MBIs was also consistent in mobile applications (apps)‐based interventions in various populations (Coulon et al., 2016; Heeter et al., 2017; Kubo et al., 2019; Mak et al., 2018; Rosen & Potter, 2018; Yang et al., 2018). Studies demonstrated that mindfulness meditation apps exerted positive psychological effects on women diagnosed with breast cancer (Kubo et al., 2019; Rosen & Potter, 2018), healthcare professionals (Heeter et al., 2017), students (Yang et al., 2018) and self‐selected internet users (Howells et al., 2016). The duration and intensity of these programmes varied from daily 10‐min sessions over 8 weeks (Kubo et al., 2019), 10‐ to 20‐min daily courses over a month (Yang et al., 2018) and courses as brief as 10 min of mindfulness exercises a day for 10 days (Howells et al., 2016).

Similarly, MBI programmes disseminated via instant messaging apps yielded positive mental health outcomes (Cheung et al., 2022; Li et al., 2022). Two Hong Kong studies delivered an intervention package consisting of 21 days of daily mindfulness exercises, including one 10‐ to 15‐min audio clip and one psychoeducational article. They provided empirical evidence that MBIs delivered through text messaging effectively improved distress related to pain, sleep and eating (Li et al., 2022) and subjective well‐being for parents (Cheung et al., 2022). The latter study that targeted parents resulted in a significant improvement in their well‐being; however, it did not lead to a notable reduction in parental stress and did not incorporate elements of ‘mindful parenting’.

The term ‘mindful parenting’ was first used to describe intentionally bringing here‐and‐now nonjudgmental attention to parenting, children and the family (Bögels & Restifo, 2014). In the past decade, mindful parenting was specifically adapted from MBIs to the context of parenthood (Kabat‐Zinn & Kabat‐Zinn, 1998). Meta‐analytic studies showed that mindful parenting courses successfully reduced stress for parents of children of different ages in both online and offline settings (Caetano et al., 2024). However, to our knowledge, no research has evaluated the effectiveness of instant messaging‐based interventions in reducing parenting stress through a programme that incorporates mindful parenting elements. The present studies aimed to address this research gap.

Substantial research has demonstrated that low socioeconomic status (SES) has adverse psychological effects on children and families (Chaudry & Wimer, 2016; Kaiser et al., 2017; Peverill et al., 2021). According to the family stress model, socioeconomic hardships exacerbate child maladjustment primarily through psychological distress among parents, poor interparental relationships and disruption of parenting (Masarik & Conger, 2017). Healthcare expenses further divert already limited disposable income from the mental health needs of families. The high cost of mindfulness‐based programmes may be prohibitive for socioeconomically disadvantaged families, as they may not have the financial resources to invest in such interventions.

In Hong Kong, many socioeconomically disadvantaged parents may not be aware of their own mental health difficulties and the resources available, or they may not know how to navigate the healthcare system, leading to the underutilisation of services (Tan et al., 2023). Cultural stigma also served as a barrier that may deter individuals with mental health challenges from seeking help, particularly among those from lower socioeconomic backgrounds (Tan et al., 2023). Public mental health services are often limited, with long waiting times and insufficient resources to meet the demand, disproportionately affecting socioeconomically disadvantaged families who do not have the resources to opt for private medical services (Sun et al., 2018; Tan et al., 2023).

In addition, parents' long working hours further limit their availability to participate in parenting programmes. In the UBS Price and Earnings Report 2015, out of 71 cities surveyed, Hong Kong had the longest working hours (2606 per year) in the world (UBS Prices and Earnings 2015 study: Zurich, Geneva and New York City are the most expensive cities in the world|UBS Global, 2015). Many socioeconomically disadvantaged parents in Hong Kong had demanding work schedules and multiple responsibilities, limiting their ability to engage in face‐to‐face and lengthy interventions. Balancing work and family obligations often resulted in prioritising immediate needs over participation in extended self‐care programmes. As socioeconomically disadvantaged parents often prioritise work and financial stability over participation in self‐care or parenting programmes, it was suggested that brief and effective interventions are more appealing to them (Kaltman et al., 2016; Levy & O'Hara, 2010; Roach et al., 2019). To encourage socioeconomically disadvantaged families to participate in our programme, we actively engaged and consulted parents from low‐income families in the design of our intervention so that we can better address the potential barriers that may prevent them from participation. This engagement in the design phase was consistent with research showing that higher user involvement generally leads to increased satisfaction, improved quality, greater acceptance and adherence to interventions (Ives & Olson, 1984). This practice also aligned with the APA principle of population health, which emphasises adapting and evaluating evidence‐based programmes to meet subpopulation groups' needs to ensure equitable access and implementation (Dodge et al., 2024).

## THE PRESENT STUDIES

Two studies with randomised control trials were conducted. They investigated whether participating daily for 12–17 min in a 14‐day instant messaging‐based parenting programme can primarily reduce parental stress. Secondary outcomes of whether the intervention can facilitate mental well‐being, supportive parenting and decreased hostile parenting behaviours were also examined.

In the parenting literature, two major sources of parenting stress were suggested. The first was associated with child behaviours, and the second was parents' perception of parental competence (Luster & Okagaki, 2006; Mash & Johnston, 1990; Nieuwboer et al., 2013). A local study showed that parents who perceived their children as more demanding, less acceptable and less reinforcing had more parenting stress (Kwok & Wong., 2000). Parenting self‐efficacy moderated the effects of parenting stress on parents' mental health (Kwok & Wong., 2000). In mindful parenting, parents learned to apply the skills of mindfulness and compassion to (1) elicit positive changes in how they perceive their children, as well as their parenting roles, and (2) improve parental regulation of stress and reduce impulsive parenting reactions (Bögels & Restifo, 2014; Potharst et al., 2021). Although this course did not represent a direct adaptation of the mindful parenting programme, these mindful parenting principles and elements were incorporated. The intervention also aimed to enhance parenting self‐efficacy by incorporating practical skills to minimise parenting conflict, in light of the escalating child abuse rates in Hong Kong during the pandemic when the studies were conducted (Child Abuse in Hong Kong Escalating as Pandemic Sees Children and Stressed Parents Spending More Time at Home, Expert Says|South China Morning Post, n.d.). As families spent more time at home, stress experienced by both children and parents increased. The programme referenced online parenting resources (Cluver et al., 2020; Ward et al., 2020) and emphasised principles of nonviolent communication (Rosenberg & Gandhi, 1999). It included practical techniques for expressing emotions to manage negative feelings and effectively resolving conflicts. It was hypothesised that learning parenting skills and practicing mindful, self‐compassionate exercises would reduce parental stress. Study 2 replicated Study 1 using the same experimental design and intervention.

## STUDY 1

### Method

#### Ethics approval and trial registration

The first and second authors’ academic institution's, Survey and Behavioral Research Ethics Committee approved the present study, and the trials were registered at Clinicaltrials.gov.

### Design

We consulted parents from low SES families about possible barriers that deter their participation and ways to tackle them to promote participation and adherence in the design of the intervention by collaborating with Caritas, an organisation with 55 years of experience in providing social services to underprivileged communities in Hong Kong (Caritas Hong Kong, n.d.). Staff from two Caritas Community Centres gathered insights from parents in low‐income families during their regular meetings. Feedback included preferences regarding programme design and delivery, such as optimal course length, acceptable daily time commitments and preferred delivery methods. They indicated a preference for brief as well as flexible daily commitments (i.e. around 15 min and flexible training time) and suggested that the course duration not exceed 2 weeks. Low‐income parents were familiar with using WhatsApp and Signal, and they preferred these platforms as suitable means for delivering course materials. Additionally, it was brought to our attention that some of these families resided in subdivided flats, highlighting the need to adjust the format of the exercises in this course. In Hong Kong, low‐income families often face significant housing challenges due to the high cost of living and limited availability of affordable, government‐subsidised housing (Huang, 2017). The average waiting time for public housing in the Year 2021/2022 was 6.1 years (Authority, 2022). As low‐income families await government housing, many of them rent subdivided flats, which are typically smaller, partitioned units within larger apartments. Living areas of subdivided units ranged approximately from 4.6 to 10.8 m^2/capita, with an average of 6.2 m^2/capita (Tim Wong, 2018). Despite the cramped living conditions, subdivided flats were opted for primarily because of lower rent and may be closer to the city center, which reduced travel time to workplaces/schools. Our target users expressed concerns about the challenges they might face with exercises that require more space, such as mindful yoga. In response, we designed all course exercises to be practiced while seated, ensuring accessibility regardless of spatial limitations should the participants choose to practice at home.

#### Participants

This study targeted parents with at least one child in nursery, kindergarten, or primary school. Inclusion criteria were selected based on local research findings that indicated elevated stress levels among parents with children from primary school or below, released shortly before the study was conducted (Low, 2020; ‘Research: 33% Parents under High Stress in Times of Pandemic,’ 2020). Other eligibility criteria for participation included the ability to understand Cantonese and having access to text messaging apps, WhatsApp, or Signal.

A priori power analyses were conducted to determine the required sample size for detecting a time × condition interaction effect using ANOVA with repeated measures, focusing on within–between interactions across three time points (pre‐, post‐ and follow‐up assessments) and two conditions (experimental and waitlist control groups). Based on a review and meta‐analysis of randomised controlled trials of online MBIs aimed at improving mental health (Spijkerman et al., 2016), we anticipated a small to moderate effect size. With an effect size of 0.17 (Cohen's f), an alpha level of 0.05 and a desired power level of 0.80, our calculations indicated that a minimum sample size of 70 participants was necessary to ensure sufficient statistical power to detect an interaction effect. Considering a weighted average dropout rate of 31% of participants who discontinued Internet‐based treatment for psychological disorders (Melville et al., 2010), we aimed for a minimum sample size of 101 participants at follow‐up. Since our project's goal was also to provide psychological support for parents, we did not impose a cap on the number of participants to ensure that more parents could benefit from the intervention. This decision was made considering that a larger sample size is likely to yield more stable outcomes.

#### Procedure

Recruiting posters were distributed via social media platforms and to low‐income families served by two local community centres that were collaborative partners in the project. We attempted to recruit participants with limited financial resources to ensure that the intervention could benefit a broader range of people in need. All participants were recruited in the period from August 10 to 27, 2021. Parents were asked to enroll in this study via messaging a designated WhatsApp or Signal account, the two most commonly used mobile applications in Hong Kong (Explainer: Why People Are Leaving WhatsApp for Signal and Telegram ‐ Young Post|South China Morning Post, n.d.).

A link directing to the online pre‐intervention questionnaire was sent to all participants via WhatsApp or Signal 1 day after the programme enrollment deadline. The pre‐intervention questionnaire on Qualtrics excluded parents who did not have at least one child in nursery, kindergarten, or primary school from participating in the study. Participants who met all inclusion criteria were required to complete the questionnaire and provide informed consent 2 days after receiving the link. Participants who had not completed the pre‐experiment questionnaire were excluded from the study. All enrolled participants were randomly assigned to either the intervention or waitlist control groups, using a block design with a block size of 6. The waitlist group was informed that they had been assigned to a class scheduled for a month later due to the original course being full. Staff from the Caritas Community Centre handled recruitment and delivered the intervention, while the authors handled the randomisation and data analysis.

All participants took part in the same programme intervention, but their starting times differed based on the condition to which they were assigned, with the experimental group receiving the intervention immediately, and the waitlist control group receiving the intervention after they completed all the questionnaires for this study. All participants were asked to complete the post‐experiment questionnaire on Qualtrics 2 weeks after completion of the pre‐experiment questionnaire. One hundred thirty‐two (60%) participants completed the post‐experiment questionnaire.

**FIGURE 1:**
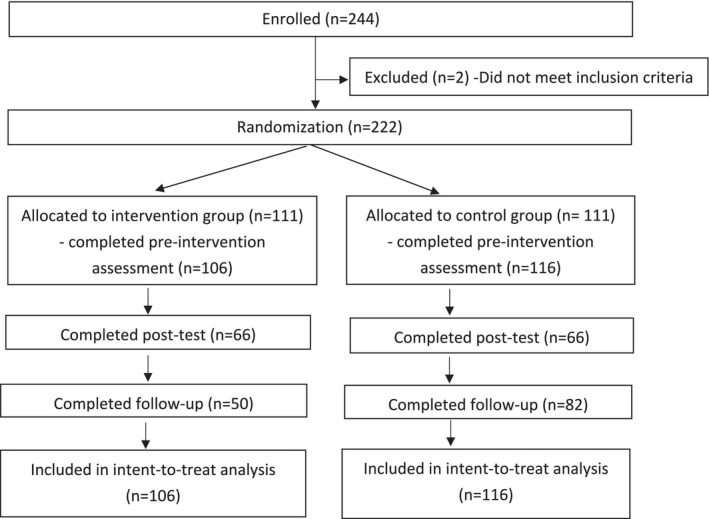
Flowchart of participants: recruitment and retention.

All participants were invited to complete a follow‐up questionnaire on Qualtrics 2 weeks after the post‐experiment questionnaires to assess if changes can be maintained. One hundred thirty‐eight (62.7%) participants completed the follow‐up experiment questionnaire. Figure 1 summarises the CONSORT flowchart for participant recruitment.

#### Intervention

Daily instructional recordings were delivered to the participants via two instant messaging apps, WhatsApp and Signal for 2 weeks for a total of 14 recordings. A welcoming audio was sent to the participants 1 day before the start of the programme. To engage parents in the programme effectively, participants were asked to practice the daily exercises at an undisturbed time and location of their choosing. They were encouraged to reach out if they experienced any difficulties or discomfort during the course.

Daily recordings of the programme lasted from 12 to 17 min. Each recording had the same format that included (1) a daily theme, (2) an exercise echoing the theme, (3) a suggested home exercise for practice and (4) an invitation for participants to give an optional response to share their meditation experience at the end of the recording. Text reminders were sent out to prompt participants to listen to the recordings if they did not respond for three consecutive days.

The first author designed and prepared all audio recordings under the guidance of the second author, who is a professor at the Psychology Department of the Chinese University of Hong Kong who practices mindfulness and does mindfulness research. When the intervention was developed, the first author was a graduate student in clinical psychology. She received 4 years of meditation training, including attending three 7‐ to 10‐day traditional meditation retreats. She co‐led mindfulness‐based programmes and groups at the Caritas Community Centre with an experienced practitioner during her years of training. These programmes and groups included parents from a range of socioeconomic backgrounds. Exercises and homework introduced in the intervention were adapted from these experiences including mindfulness daily practices (Bays, 2011), exercise adapted from nonviolent communication (Rosenberg & Gandhi, 1999), as well as mindfulness‐ and compassion‐based exercises from the Mindful Parenting protocol (Bögels & Restifo, 2014).

The first week of the programme focused on enhancing parents' ability to regulate stress by anchoring participants in the present and cultivating a curious, open and nonjudgmental awareness of their internal states, including emotions, thoughts, bodily sensations and action tendencies. We selected practices from the Mindful Parenting protocol that shared this aim and also other exercises that could be easily integrated into daily routines (Bays, 2011). Parents were encouraged to apply this mindful awareness during interactions with their children (Bögels & Restifo, 2014). In the second week, the programme fostered mindful compassion in the face of stressful parenting experiences. Parents were encouraged to adopt a mindful attitude during challenging interactions with their children and to accept, with compassion, the difficult emotions they encounter. They practiced delivering loving‐kindness to themselves and their children. They were also guided to identify, acknowledge and express their own emotional needs with compassion during moments of rupture and to extend it to their children (Bögels & Restifo, 2014; Rosenberg & Gandhi, 1999). Table S1 provides a summary of themes and exercises included in our intervention.

#### Measures

All questionnaires were measured in Chinese.

### Demographic information

Participants' demographic information, including age, gender and education level, was obtained. Low‐income families were identified in the present study if their reported household income did not exceed 75% of the median family income with respect to the number of household members and were eligible to apply for government financial assistance (Working Family and Student Financial Assistance Agency ‐ Working Family Allowance Scheme—Scheme Characteristics—Working Family Allowance Scheme—Scheme Characteristics, n.d.). In this study, around one third (34%, *n* = 78) of the parents corresponded to socioeconomically disadvantaged groups.

### Adherence, programme difficulty and general comments

The participants in the intervention group were asked to indicate their adherence to the training programme in the post‐intervention questionnaire in a continuous format from 0 to 14 days. They were also asked to respond to one activity rating question, that is, ‘To what extent did you find the programme difficult to complete? (1, *Not at all* to 5, *Extremely*)’. They were asked to explain the difficulties encountered. They were also invited to give overall comments on the programme.

### Follow‐up practice

In the follow‐up questionnaire, the intervention group was asked to report the number of days of mindfulness practice they have engaged in after completing the online course (0, *Never*; 1, *Once per week*; 2, *Twice or thrice a week*; 4, *Four to six times a week*; 5, *Seven to nine times a week*; and 6, *More than 10 times per week*).

#### Primary outcome

##### Parental Stress Scale (PSS)

The 16‐item Chinese version of the PSS (Leung & Tsang, 2010) was used to measure perceived parental stress on a 6‐point Likert scale ranging from 1 (*Strongly agree*) to 6 (*Strongly disagree*). Berry and Jones developed PSS to measure parental feelings and experiences regarding rewards, satisfaction, controllability and stress (Berry & Jones, 1995). Two items were deleted from the original version due to low item‐total correlation in the Chinese version (Leung & Tsang, 2010). Items included ‘I am happy in my role as a parent’ and ‘The major source of stress in my life is my child(ren)’. In this study, Cronbach's alpha was 0.87.

#### Secondary outcomes

##### 5‐Item World Health Organisation Well‐being Index (WHO‐5)

Global mental well‐being was measured using the 5‐item WHO‐5 (Topp et al., 2015). Participants were asked to indicate their feelings over the last 2 weeks on a 6‐point Likert scale from 0 (*At no time*) to 5 (*All the time*). Items included ‘My daily life has been filled with things that interest me’ and ‘I have felt cheerful and in good spirits’. The Chinese version of this scale was used (Topp et al., 2015). In the present study, Cronbach's alpha was 0.87.

##### Interpersonal Mindfulness in Parenting (IM‐P) Scale

The 31‐item IM‐P scale measured parents' self‐reported engagement in mindful parenting (Duncan et al., 2009). This instrument measured five dimensions: (1) Five items tapped into listening with full attention (LFA) to the child (e.g. ‘I find myself listening to my child with one ear because I am busy doing or thinking about something else at the same time’); (2) Seven items tapped into nonjudgmental acceptance (NJA) of the self and the child, (e.g. ‘When things I try to do as a parent do not work out, I can accept them and move on’); (3) Six items tapped into emotional awareness of the self and the child (EA) (e.g. ‘It is easy for me to tell when my child is worried about something’); (4) Six items tapped into self‐regulation (SR) in the parenting relationship (e.g. ‘When I am upset with my child, I notice how I am feeling before I take action’); and (5) Seven items tapped into compassion for the self and the child (C) (e.g. ‘When I do something as a parent that I regret, I try to give myself a break’) (Duncan et al., 2009).

Participants were asked to respond on a 5‐point rating scale from 1 (*Never true*) to 5 (*Always true*). Higher scores reflect more mindfulness in parenting. Adequate validity was established in the subscales, and satisfactory validity was established for the full scale and subscales (Beer et al., 2013). The IM‐P was shown to be sensitive to intervention change (Coatsworth et al., 2010, 2015). The Chinese version of IM‐P was adopted from Lo et al. (Lo et al., 2018). In this study, Cronbach's alpha was 0.86 for the full scale. Cronbach's alpha across the subscales was 0.75 for LFA; (2) 0.75 for NJA after deleting one item with low correlation; (3) 0.43 for EA, and it was excluded from further analysis due to low reliability; (4) 0.71 for SR; and (5) 0.76 for C after deleting three items with low correlation.

##### Parent Behaviour Iventory (PBI)

The PBI was used to assess changes in parenting behaviour. PBI is a brief measure of parenting behaviour used with parents of preschool‐aged and young school‐aged children (Lovejoy et al., 1999). The PBI's two independent scales, supportive/engaged and hostile/coercive parenting, showed sufficient content validity and internal consistency (Lovejoy et al., 1999). Participants were asked about how they and their child(ren) generally got along in the past 2 weeks on a 6‐point Likert scale that ranged from 1 (*Never true*) to 6 (*Always true*). Back‐translation procedures were used to translate the English version into Chinese (Brislin, 1970). In this study, Cronbach's alpha was 0.80 for the *Hostile/Coercive* scale and 0.88 for the *Supportive/Engaged* scale.

#### Data analysis

All analyses were performed using SPSS Version 22 (SPSS, Inc., Chicago, Illinois, USA). One‐way ANOVA (for continuous variables) and chi‐square tests (for categorical data) were conducted to explore baseline differences between the participants who retained and dropped out from the study, as well as between the intervention and waitlist control groups.

Linear mixed model (LLM) analyses examined the time by group interaction effects on the measured variables. With reference to the intent‐to‐treat principle, all the missing data were addressed using maximum likelihood estimation. This was to avoid bias in the analysis that included only participants who completed the intervention programme (Shah, 2011). In the LLM analyses, PSS, WHO‐5, IM‐P and PBIs were entered as dependent variables separately. Conditions (i.e. experimental and waitlist control group) and time (pre‐, post‐ and follow‐up measurements) were considered as the independent variables (IV), and the interaction term of the two IVs was also structured. Subsequently, post hoc tests were performed to examine the source of significance when a significant time by condition interaction effect was observed.

### Results

#### Participants characteristics

No significant difference between participants who retained and dropped out from the study, as well as between participants in the control group and intervention group, was found in demographic information and baseline scores on all the outcome variables. No significant baseline difference was found between the socioeconomically disadvantaged and advantaged groups across all outcome measures. Table 1 shows the demographic data and baseline characteristics of the participants.

**TABLE 1:** Demographic data and baseline characteristics of the participants.

	Study 1 (*N* = 222)			Study 2 (*N* = 271)		
Characteristics	Intervention group mean/percentage (*SD*)	Waitlist control group mean/percentage (*SD*)	Group difference *p*‐value (two‐tailed)	Intervention group mean/percentage (SD)	Waitlist control group mean/percentage (SD)	Group difference *p*‐value (two‐tailed)
**Age** (in years)	38.73 (5.33)	39.56 (4.70)	.227	39.79 (4.64)	40.64 (5.31)	.164
Gender			.059			.779
Men	12.4%	5.2%		10.5%	11.6%	
Women	87.6%	94.8%		89.5%	88.4%	
Education			.080			.348
Primary	0.95%	0.87%		0.75%	1.45%	
Secondary	30.47%	19.13%		14.29%	21.01%	
Higher education	17.14%	15.65%		21.80%	14.49%	
Bachelor's	31.42%	49.57%		39.10%	42.03%	
Master's or above	20%	14.78%		24.06%	21.01%	
Marital status			.336			.634
Married	90.48%	93.04%		92.97%	89.47%	
Separated/divorced	7.62%	5.21%		6.02%	8.59%	
Widowed	0.95%	0%		0%	0%	
Single	0.95%	0%		0.78%	1.56%	
Cohabit	0.95%	1.74%		0%	0.75%	
Employment			.864			.588
Full‐time	44.56%	40%		51.13%	55.07%	
Part‐time	15%	14.13%		9.42%	15.04%	
Unemployed	0%	1%		0.75%	1.45%	
Homemaker	39.13%	42%		33.08%\	34.05%	
Socioeconomic status			.895			.308
Low‐income family	35.64%	34.78%		32.28%	26.51%	
Non‐low‐income family	64.36%	65.22%		67.72%	73.48%	

#### Adherence

In the intervention condition, of the 66 participants who completed the post‐course questionnaire, 40 participants (60.6%) completed 13 or 14 days of the audio recordings from the training programme. Fifty‐nine participants (89.3%) completed at least 7 days. The average self‐reported completed days of the programme was 11.83 (*SD* 3.56). Table S2 provides a summary of the self‐reported days of recordings completed by participants.

Research suggested that greater engagement in formal mindfulness practice was associated with enhanced levels of self‐reported mindfulness, which in turn was linked to improvements in well‐being (Carmody & Baer, 2009). Thus, the self‐reported number of completed audio recordings was entered as a covariate to investigate the relationship between adherence and the programme's effectiveness. No significant three‐way interaction effect was found when the number of completed days of recording was treated as a covariate in the previous LLM analysis, with primary and other secondary outcomes included as the dependent variable. No significant relationship was found between participants' demographics, including SES, and their completed days of recording.

At follow‐up assessment, that is, 14‐day post‐intervention, more than half of the participants (52.4%) indicated that they did not engage in any mindfulness practice (37.8%, *n* = 31) or that they practiced once a week (14.6% *n* = 12). Table S3 provides a summary of the self‐reported days of mindfulness practice completed at follow‐up assessment.

#### Programme difficulty

Overall, 40.9% of the participants found the programme very easy or easy to complete on the one hand. On the other hand, 37.8% of the participants found it difficult or very difficult to complete. Participants indicated the latter reasons included difficulty finding time (72.3%) and a quiet space (12%) to listen to the recordings. The remaining 21.2% of participants had no comments about the programme's difficulty. Table S4 summarises self‐reported difficulty ratings, while Table S5 outlines the reasons participants found the programme difficult to complete. Chi‐square tests of independence showed that there were no significant relationships between participants' demographics, including SES, and their ratings of programme difficulty.

#### General comments

Participants were asked to leave optional comments on the programme; 79.6% of the comments felt positive about their experience. While over 50% of them did not specify the reason behind it, 14% indicated that the intervention helped them relax. Others (10.2%) indicated their difficulty in completing the programme. For example, two participants (4%) had difficulty allocating time for daily recordings. Some participants (14%) suggested improvements, such as changing the audio speed. Three participants (6%) wanted to lengthen the programme. Table S6 summarises these general comments.

#### Intervention effect

For the intervention effects, no significant time (pre‐, post‐ and follow‐up intervention) by group (intervention and control group) interaction was found in all outcome variables.

To examine the effect without accounting for the sustained effect in follow‐up, the same analyses were rerun with only pre‐ and post‐assessments. Significant time by group interaction was only found in parental stress *F*(1,124.91) = 5.18, *p* = .025, with a small effect size (Cohen's *d* = .30). Table 2 summarises the results. Post hoc test showed that compared to baseline, participants from the intervention group had a significant decrease in parental stress post‐intervention; the mean difference between the two time points was −2.72, *F*(1,124.849) = 11.48, *p* = .001, 95% CI [−4.31, −1.31], Cohen's *d* = .28. No significant effect was observed in the waitlist control group; the mean difference between the two time points was −0.13, *F*(1,124.926) = 0.26, *p* = .872, 95% CI[−1.71, 1.46].

**TABLE 2:** Linear mixed model analysis for Study 1 in all outcome measures over two time points.

	Pre‐intervention	Post‐intervention	Interaction effect		Condition effect		Time effect	
	Mean	Mean	F (df)	*p*‐Value	F (df)	*p*‐Value	F (df)	*p*‐Value
Parental stress (PSS)			5.183 (1,124.91)	.025	.026 (1, 214.99)	.872	6.28 (1, 124.91)	.014
Intervention	49.94	47.22						
Waitlist	48.44	48.31						
Mental well‐being (WHO‐5)			.577 (1,125.71)	.45	1.66 (1, 195.39)	.20	21.38 (1,125.71)	<.001
Intervention	15.28	17.02						
Waitlist	16.26	17.51						
Hostile parenting in PBI								
Intervention	33.46	32.00	1.49 (1,118.92)	.23	.66 (1,201.38)	.42	4.81 (1,118.92)	.030
Waitlist	33.64	33.22						
Supportive parenting in PBI			.072 (1,117.61)	.79	1.27 (1,199.91)	.26	.48 (1,117.61)	.827
Intervention	46.70	46.50						
Waitlist	47.59	47.57						
Interpersonal mindfulness in parenting (IM‐P)			2.31 (1,129.38)	.13	.76 (1,210.31)	.38	1.52 (1,129.38)	.22
Intervention	73.26	74.88						
Waitlist	73.10	72.93						

No significant three‐way interaction effect was found when the difficulty rating was treated as a covariate in the LLM analysis of primary outcome variable changes. Regression analysis was conducted using programme difficulty (range: 1–5) to predict programme completion days and outcome changes. Results did not show a significant relationship between difficulty and completion ratings. To examine whether difficulty ratings impacted outcome changes, participants who rated the programme as easy or very easy and those who rated it difficult or very difficult were dummy‐coded into two groups for further comparison. The ‘no comment group’ was treated as the reference group. Difficulty ratings did not significantly influence stress reduction after baseline scores were controlled.

## STUDY 2

### Method

#### Design

Design was identical to Study 1.

#### Participants

The target participants and data collection process were identical to Study 1. Participants were recruited from 13 to 20 January 2022, 334 parents signed up. A total of 271(81.6%) valid pre‐experimental questionnaires were received. Since the actual recruitment met the minimum target of 101 at follow‐up, our final sample was deemed adequate for evaluating the measured outcomes.

#### Ethics approval and trial registration

Ethics approval and clinical trial registration were identical to Study 1.

#### Procedure

**FIGURE 2:**
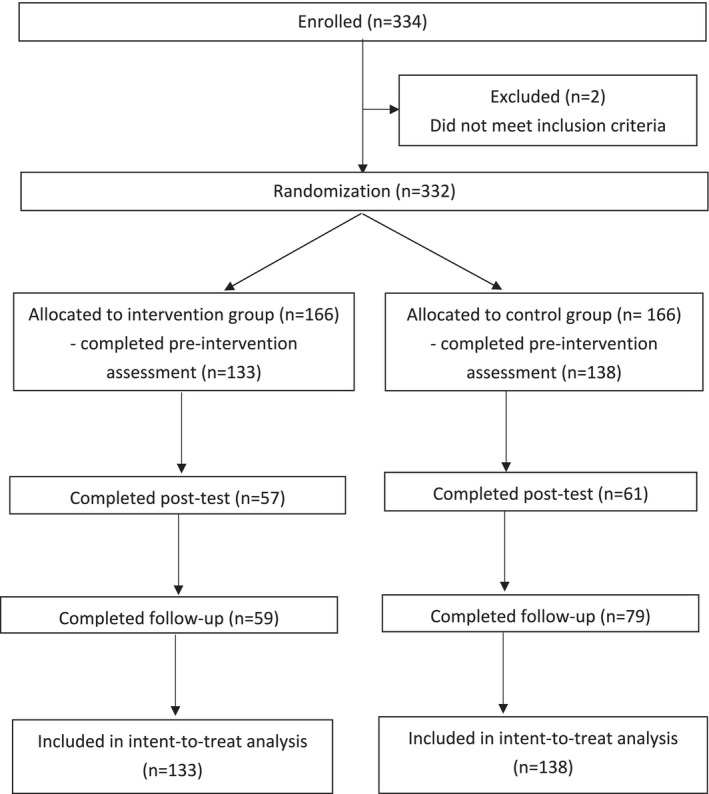
CONSORT flowchart of participants: recruitment and retention.

The procedure was identical to Study 1; 118 (43.5%) and 138 (50.9%) participants completed the post‐ and follow‐up experiment questionnaire, respectively. Figure 2 summarises the CONSORT flowchart for participant recruitment.

#### Intervention

The intervention used was identical to Study 1.

#### Measures

The measures used were identical to Study 1.

#### Primary outcome

##### PSS

In this study, Cronbach's alpha was 0.87.

#### Secondary outcomes

##### WHO‐5

In this study, Cronbach's alpha was 0.90.

##### IM‐P

In this study, Cronbach's alpha was 0.86 for the full scale. Cronbach's alpha across the subscales was (1) 0.75 for LFA; (2) 0.77 for NJA after deleting one item with low correlation; (3) 0.4 for EA and it was excluded from further analysis due to low reliability; (4) 0.75 for SR; and (5) 0.77 for C after deleting three items with low correlation.

##### PBI

In this study, Cronbach's alpha was 0.78 for the Hostile/Coercive scale; 0.92 for the Supportive/Engaged scale.

#### Data analysis

All analyses performed were identical to Study 1.

### Results

#### Participants characteristics

No significant difference between participants who retained and dropped out from the study, as well as between participants in the control group and intervention group, was found in demographic information and baseline scores on all the outcome variables. No significant baseline difference was found between the socioeconomically disadvantaged and advantaged groups across all outcome measures. Table 1 shows the demographic data and baseline characteristics of the participants. Around one third (29%, *n* = 78) of the parents corresponded to socioeconomically disadvantaged groups.

#### Adherence

In the intervention group, of the 57 participants who completed the post‐intervention questionnaire, 26 participants (47.6%) completed all 13 or 14 days of the audio recordings from the training programme. Forty‐two participants (73.7%) completed at least 7 days. The average number of self‐reported completed days in the programme was 10.01 (*SD* 4.47). Table S2 provides a summary of the self‐reported days of recordings completed by participants. No significant three‐way interaction effect was found when the number of completed days of recording was treated as a covariate in the previous LLM analysis, with primary and secondary outcomes entered as the dependent variable. Chi‐square of independence tests did not reveal any significant relationship between participants' demographics, including SES, and their completed days of recording.

At the follow‐up assessment, which is 14 days post‐intervention, 60% of participants indicated that they did not engage in any mindfulness practice (27.42%, *n* = 17) or only practiced at least once a week (32.26%, *n* = 20). Table S3 provides a summary of the self‐reported days of mindfulness practice at the follow‐up assessment.

#### Programme difficulty

Overall, 36.7% of the participants find the programme very easy or easy to complete. However, 43.5% of the participants find it difficult or very difficult to complete. Participants indicated that the latter reasons included difficulty finding time (58.3%) and quiet space (16.7%) to listen to the recordings. The remaining participants had no comment regarding the programme's difficulty. Table S4 summarises self‐reported difficulty ratings, while Table S5 outlines the reasons participants found the programme difficult to complete. The chi‐square of independence test revealed no significant relationship between participants' demographics, including SES, and their ratings of programme difficulty.

#### General comments

Forty‐seven participants left optional overall comments on the programme; 72.3% of the comments felt positive about their experience or shared elements of the programme they appreciated. Others indicated their difficulty in completing the programme, and some suggested improvements. Three participants (6%) indicated difficulty in finding time to complete the programme. Three participants (6%) found the audio too long. One participant reported having encountered technical difficulties that hindered his or her engagement. Table S6 summarises these general comments.

#### Intervention effect

For the intervention effects, no significant time (pre, post and follow‐up intervention) by group (interventional and control group) interaction was found in all outcome variables.

To examine the effect without accounting for the sustained effect in follow‐up, the same analyses were rerun with only pre‐ and post‐assessments. For the primary outcome, a significant time × group interaction was found in parental stress with a small effect size *F*(1,116.72) = 4.144, *p* = .044, Cohen's *d* = 0.25. Post hoc tests indicated that compared to baseline, participants from the intervention group had a significant decrease in parental stress post‐intervention; the mean difference between the two time points was −2.12, *F*(1,116.28) = 8.43, *p* = .004, 95% CI[−3.56, −.67], Cohen's *d* = .23. No significant difference was observed in the waitlist control group; the mean difference between the two time points was −.021, *F*(1,117.16) = 0.001, *p* = .978, 95% CI[−1.46, 1.42].

For secondary outcomes, a significant time x group interaction was only found in the Supportive/Engaged Scale of the PBI *F*(1,106.88) = 5.29, *p* = .023, Cohen's *d* = .3. Post hoc tests indicated that compared to baseline, participants from the control group had a significant decrease in supportive parenting behaviours post‐intervention; the mean difference between the two time points was −1.36, *F*(1,107.25) = 6.92, *p* = .01, 95% CI [−2.45, −.34], Cohen's *d* = .21. No significant change was observed in the intervention group; the mean difference between the two time points was 0.29, *F*(1,106.45) = .33, *p* = .565, 95% CI[−0.571, 1.23]. No significant time by group interaction was found in other secondary outcome variables, nor the subscales of the IM‐P Scales. Table 3 summarises these results.

**TABLE 3:** Linear mixed model analysis for Study 2 in all outcome measures over two time points.

	Pre‐intervention	Post‐intervention	Interaction effect		Condition effect		Time effect	
	Mean	Mean	F (df)	*p*‐Value	F (df)	*p*‐Value	F (df)	*p*‐Value
Parental stress (PSS)			4.144 (1,116.72)	.044	.191 (1, 247.42)	.663	4.31 (1,116.72)	.040
Intervention	51.48	49.36						
Waitlist	49.91	49.89						
Mental well‐being (WHO‐5)			.063 (1,121.35)	.80	.003 (1,220.51)	.96	19.04 (1,121.35)	<.001
Intervention	15.08	16.62						
Waitlist	15.02	16.75						
Hostile parenting in PBI			.562 (1,119.86)	.46	1.23 (1,238.90)	.268	10.65 (1,119.86)	.001
Intervention	34.28	32.62						
Waitlist	34.81	33.76						
Supportive parenting in PBI			5.29 (1,106.88)	.023	3.20 (1,236.56)	.075	2.26 (1,106.88)	.14
Intervention	46.25	46.55						
Waitlist	45.47	44.08						
Interpersonal mindfulness in parenting (IM‐P)			1.542 (1, 118.70)	.22	.33 (1,246.95)	.57	8.57 (1,118.70)	.004
Intervention	71.59	74.13						
Waitlist	71.64	72.67						

No significant three‐way interaction effect was found when the difficulty rating was treated as a covariate in the LLM analysis of primary outcome variable changes. Regression analysis was run using programme difficulty (range: 1–5) to predict programme completion days and outcome changes. It did not reveal a significant relationship between difficulty ratings and the days of audio completed. To examine whether difficulty ratings impacted outcome changes, participants who rated the programme as easy or very easy and those who rated it difficult or very difficult were dummy‐coded into two groups for further comparison. The ‘no comment group’ was treated as the reference group. Difficulty ratings were not significantly associated with stress reduction after baseline scores were controlled.

#### Adverse effect

One participant reported that engaging in meditation exercises triggered traumatic childhood memories. She was advised to discontinue the project. She was offered supportive counselling.

## GENERAL DISCUSSION

### Results

Two studies used a randomised controlled trial design to investigate the effects of a parenting programme delivered via instant messaging apps on a community sample of parents. Approximately one third of the participants were socioeconomically disadvantaged. Study 2 was a replication of Study 1. The intervention significantly reduced parenting stress in both studies, with a small effect size. However, the intervention did not yield significant improvements in secondary outcomes, including interpersonal mindfulness in parenting, hostile parenting behaviours and overall mental well‐being. Although it was encouraging to observe significant stress reduction after investing only around 15 min per day over 14 days, the intervention effects were not sustained at the 2‐week follow‐up assessment.

Previous studies showed that mindfulness‐based training successfully diminished not only parents' parental stress but also improved mindful parenting, overall well‐being and impulsive parenting reactions and that these gains remained stable at 1‐month follow‐up (Boekhorst et al., 2021; Bögels & Restifo, 2014; Corthorn, 2018; Kabat‐Zinn & Kabat‐Zinn, 1998). However, these trainings are typically conducted face‐to‐face and last 3 hours over 8–10 consecutive weeks. It is plausible that a more intensive intervention (e.g. lengthening intervention days or length of audios) may be needed to yield positive effects on these variables and to sustain outcome gains. For example, local research demonstrated that participants who experienced a 21‐day intervention package, which included one 10‐ to 15‐min audio clip and one psychoeducational article delivered via WhatsApp, sustained psychological improvements at 1‐month follow‐up (Cheung et al., 2022; Li et al., 2022). It will be necessary to conduct further studies to determine which programme length may enhance participants' commitment and engagement, given that more than half of those who deemed this course difficult to complete attributed it to a lack of time. As other local research has shown, while parents acknowledge the potential benefits of mindfulness for reducing stress and improving well‐being, the practical challenges of integrating these practices into their busy lives often prevent them from participating (Lo et al., 2016).

Additionally, it is worth noting that one of the aforementioned daily 10‐ to 15‐min, 21‐day online interventions also targeted parents (Cheung et al., 2022). While it effectively improved overall well‐being, it did not reduce parental stress. In contrast, the current project significantly lowered parenting stress but did not enhance overall well‐being. A key distinction between the two interventions was that the current one incorporated mindful parenting principles and elements that encouraged participants to apply mindful compassion‐based skills within their parenting context (Bögels & Restifo, 2014). For instance, parents were invited to adopt a mindful attitude during their everyday interactions with their children, as well as to practice mindful self‐compassion during stressful situations. These mindful parenting components may be essential for reducing stress in the parenting context, especially within a brief online programme lasting approximately 15 min per day for 2 to 3 weeks. However, further research is needed to identify the minimum or optimal proportion of mindful parenting elements necessary for effectively reducing parenting stress in a brief programme. More importantly, if this inference is accurate and generalisable beyond parenting, it is likely that similar interventions aimed at alleviating stress related to specific roles—such as adult caregivers for older parents or supervisors in the workplace—should involve applying mindful compassion within those particular contexts.

This study used text messaging to disseminate a brief mindfulness‐ and compassion‐based parenting course that alleviated parental stress. It demonstrated that delivering services online via instant messaging was technically feasible for parents, as only two participants (0.004%) reported technical problems accessing the audio in the two studies. While developing intervention materials took time and effort, disseminating content did not require training; this enhanced the programme's overall cost‐effectiveness.

Additionally, it is plausible that allowing parents to participate in the interventions at their convenience enabled them to better manage their work schedules, childcare responsibilities and other commitments. Online interventions may also mitigate public stigma and improve attitudes toward seeking help for mental health issues (Rodríguez‐Rivas et al., 2022). The anonymity provided by online platforms may encourage individuals to seek help without the fear of being labelled or judged, which is particularly important in cultures where mental health issues are stigmatised (MacDonell & Prinz, 2017).

Our study revealed no significant difference in baseline parenting stress between low‐income families and those from higher socioeconomic backgrounds. This finding contrasts with previous studies suggesting that parents from lower SES backgrounds experience higher levels of stress compared to those from higher SES backgrounds (e.g. Martins et al., 2023). We would like to highlight that this discrepancy may stem from the timing of our studies, which were conducted during periods when most parents faced increased caregiving responsibilities due to the intermittent suspension of face‐to‐face classes in kindergartens and primary schools, driven by rising COVID‐19 infection rates (News.gov.hk, 2020; News.gov.hk, 2022). Such suspension may have increased parental stress for most parents (Achterberg et al., 2021; Cluver et al., 2020), leading to comparable levels of parenting stress regardless of SES. We believe that social disparities remain a significant concern and that future intervention designs should address the unique barriers that disadvantaged groups may encounter in the post‐COVID landscape, as these groups may experience poorer mental health outcomes due to increased stressors and limited access to resources (Chaudry & Wimer, 2016; Kaiser et al., 2017; Peverill et al., 2021).

As Dodge et al. (2024) highlighted, many ‘validated’ evidence‐based programmes may not be appropriate or effective for specific subpopulations. Similarly, in the past, limited studies on MBIs for socioeconomically disadvantaged families have been conducted (Jiga et al., 2019; Lo et al., 2019). The findings of existing MBI studies may not fully reflect the acceptance and feasibility of MBIs for socioeconomically disadvantaged families because they underrepresent this population. Our analyses showed that participants' demographic characteristics, including socioeconomic background, did not significantly influence the intervention effect, programme difficulty ratings and completion rates. This finding may reflect the positive outcome of engaging parents from socioeconomically disadvantaged families in the intervention's design process. However, additional evidence is required to confirm the intervention's effectiveness in both socioeconomically deprived and non‐deprived groups. Our study could not achieve this, as the low‐income family subgroup represents one third or less of the total sample in both studies. Conducting a separate analysis could skew the results and misrepresent the overall effectiveness of the intervention. Nonetheless, we believe that user‐centred design interventions could greatly enhance future programmes for underserved populations. By incorporating the perspectives and needs of these families, we can increase satisfaction, improve quality, and foster greater acceptance as well as adherence to interventions (Ives & Olson, 1984; Lyon & Koerner, 2016).

In addition, our programme operated under the assumption that the same intervention would be accepted by families from different economic backgrounds. However, further research is needed to determine whether certain aspects of the design may need to be tailored to specific subpopulations or whether they are suitable across different backgrounds. For instance, although our studies found no significant relationship between SES and adherence rates, there is evidence suggesting that SES can influence adherence in other health promotion programmes (Lemstra & Rogers, 2021). It remains to be explored whether incentives designed to improve adherence rates might resonate differently with parents from various socioeconomic backgrounds.

In the past, traditional parenting intervention programmes often targeted specific groups with risk factors to improve parents' coping skills and mental wellness (Eames et al., 2015; Townshend et al., 2014; Ward et al., 2020). The rising mental health challenges among youth in Hong Kong have become a significant concern in recent years, as recent studies have shown that 25% of children and adolescents have been affected by a mental disorder in the past year (Study Finds 25% of Hong Kong Children and Adolescents Affected by a Mental Disorder in the Past Year—Young Post|South China Morning Post, n.d.). A similar phenomenon was observed in other countries, that is, the likelihood that a child in the United States would experience a diagnosable mental disorder by age 18 has grown to 49.5% (Merikangas et al., 2010). Systematic reviews have demonstrated the association between child mental health problems and parental stress (Păsărelu et al., 2023). Effective interventions, such as stress management programmes, can significantly improve parents' and youth's mental health outcomes (Hoagwood et al., 2012). Timely access to advice and support for families is thus critical, especially for parents and children who are faced with a long waiting time for assessment and treatment for mental health‐related difficulties. Similar interventions may be developed to support children and young people and/or their parents while on a waiting list for child and adolescent mental health services (Valentine et al., 2024).

Researchers estimated that 36% to 50% of parents experience some level of parenting stress about parenting, child behaviour or child development (O'Connor & Madge, 2004). According to research statistics in Sweden, parenting websites are visited by hundreds of thousands of people every month, suggesting that parents, in general, also need help overcoming parenting barriers (Sarkadi & Bremberg, 2005). The Internet now offers opportunities for delivering parenting information and support resources for large groups of parents in an accessible and affordable way (Sarkadi & Bremberg, 2005; Scharer, 2005). Online parenting programmes were gradually made available, and a meta‐analysis confirmed their effectiveness (Nieuwboer et al., 2013). Digital health interventions have great potential to deliver large‐scale community support at a community/policy level in successfully overcoming structural barriers, such as time, cost and stigma, that impede individuals from obtaining mental health services (Dodge et al., 2024; Murray et al., 2016). Hopefully, smartphone technology will continue to benefit local and global communities in the future, while prioritising inclusivity and accessibility.

### Limitations

Caution should be taken when interpreting the results of this project. First, the participating sample lived in Hong Kong, a fast‐paced urban city. As Hong Kong does not have rural and remote areas like other countries, this raises questions about the transferability of the results of this programme to participants with other cultures and lifestyles.

The attrition rate in the present study was high (45% and 49% in Studies 1 and 2, respectively). No significant relationship was found between the dropped‐out group, demographics, or other measures used in this study. While attrition rates were common in Internet‐based and self‐help applications (Melville et al., 2010), future studies that aim at increasing engagement may be an undeniably meaningful but challenging topic worth exploring. For instance, adherence to the online intervention protocol was higher when participants received support through group interactions or feedback/incentives from daily/weekly recordings, compared to individual, self‐determined participation (Johnson & Goolkasian, 2022). Furthermore, the samples were predominantly (over 85%) mothers, which was consistent with recruitment challenges that parenting programme providers typically faced (Caetano et al., 2024). One plausible reason that underlined the low recruitment rate of fathers may be attributable to the differences in parental stress levels between fathers and mothers, with mothers reporting overall higher stress levels than fathers (Lo et al., 2023). Another reason may be the existing barriers for men seeking formal and informal help under stress (Cheng & Lai, 2023). Further quantitative and qualitative research will be required to confirm and explore how acceptability and user preferences may differ among sexes, age groups and other demographic characteristics and contexts. For instance, strategies may be developed and evaluated in recruiting urban fathers to increase their engagement in mental health‐related programmes (Paquette, 2004).

The study explored possible factors that may have moderated participants' outcome gains. Analyses in both studies showed that completion rate did not significantly moderate stress reduction. On the one hand, this finding aligned with previous research indicating that shorter versions of Mindfulness‐Based Stress Reduction (MBSR) are not less effective than the standard format in alleviating psychological distress (Carmody & Baer, 2009). Similarly, the correlation between the mean effect size of stress reduction and the number of in‐class hours was nonsignificant for both clinical and nonclinical samples after excluding outliers (Spijkerman et al., 2016). Further investigation is needed to better understand the optimal dosage and key elements of an intervention that may sustain its effectiveness in reducing the primary outcome. On the other hand, it is also possible that this observed relationship between outcome gains and completion rate resulted from a discrepancy between self‐reported and actual completion rates. Objective measurements using backend data from digital platforms could be used to validate self‐reports of participants' engagement in future studies. The present research also did not identify what contributed to reducing parental stress. We asked participants to indicate how many days they listened to audio, but not which days. Since each audio recording had specific themes, including stress management and parenting skills, the audio(s) they listened to may have produced different changes. Further replications of similar studies may benefit from this information to better understand the mechanism of the intervention.

Analyses from both studies revealed that programme difficulty ratings did not significantly impact completion rate or outcome gains. A previous study also found no significant association between task difficulty and outcome gains (Howells et al., 2016). It is possible that participants may be intrinsically motivated to engage with and benefit from the course material, leading to similar outcomes regardless of their perceived programme difficulty. Environmental factors, such as prompts from the instructor, may also provide cues for participants to continue their practice despite the difficulties they were met with. At the same time, we acknowledge that this project did not effectively capture the challenges individuals encountered and how this may have impacted completion rate and parental stress experienced. Given this project was completed during COVID‐19's fluctuating pandemic, personal and family infection, as well as anti‐pandemic measures implemented, may all affect participants' difficulty in completing the course. Further investigations will be needed to decipher the interplay between factors such as expectancy, valence, programme difficulty, completion rate, intervention effectiveness and engagement in the post‐COVID era.

The use of a waitlist control group posed another limitation, as it did not account for placebo effects or other nonspecific factors that may influence outcomes. Future research should consider including active control conditions to provide a more comprehensive understanding of the intervention's effects and help isolate the specific contributions of the mindfulness‐ and compassion‐based practices. Finally, the study only included a 2‐week follow‐up assessment, making it unclear what the longer term impacts on participants' lives might be. A qualitative evaluation could help address this gap by providing deeper insights into the sustained effects of the intervention.

### Conclusion

The present study effectively utilised smartphone‐based methodologies, specifically instant messaging applications, to deliver a parenting course aimed at reducing parental stress. Results supported the hypothesis that this mindfulness‐ and compassion‐based intervention could reduce parental stress with a small effect size. This project exemplifies the potential of smartphone technology to improve community well‐being.

## CONFLICT OF INTEREST STATEMENT

The authors declare that they have no known competing financial interests/conflicts or personal relationships that could have appeared to influence the work reported in this paper.

## ETHICS STATEMENT

The studies adhere to the ethics of scientific publication as detailed in the Ethical principles of psychologists and code of conduct (American Psychological Association, 2016, http://www.apa.org/ethics).

## TRIAL REGISTRATION

https://clinicaltrials.gov/study/NCT05413577 (registered date: 2022‐11‐18).

## Supporting information


**Data S1.** Table 1. Summary of intervention themes and exercises.
Table 2. Self‐reported days of recordings completed by participants.
Table 3. Participants' self‐reported mindfulness practice at follow‐up.
Table 4. Response to the extent participants find the program a difficult one to complete.
Table 5. Reasons for finding the program a difficult one to complete.
Table 6. Participants' general comments.

## Data Availability

The authors have full control of all primary data, and the data may be reviewed upon request.
