# Supplementary material for: A brief mindfulness‐ and compassion‐based parenting programme delivered via instant messaging: Results and implications from two randomised controlled trials on reducing parental stress
Source: Appl Psychol Health Well Being. 2025 Nov 29;17(6):e70094. doi: 10.1111/aphw.70094 (PMC12665100; doi:10.1111/aphw.70094)
Supplement: Supplementary file 1 — Data S1. Table 1. Summary of intervention themes and exercises. Table 2. Self‐reported days of recordings completed by participants. Table 3. Participants' self‐reported mindfulness practice at follow‐up. Table 4. Response to the extent participants find the program a difficult one to complete. Table 5. Reasons for finding the program a difficult one to complete. Table 6. Participants' general comments. [file APHW-17-0-s001.docx]

Table 1. Summary of intervention themes and exercises

| Day | Theme | Exercise and Homework | Reference |
| --- | --- | --- | --- |
| 1-2 | Cultivating an open and curious present awareness | Mindful breathing | Exercise from Mindful Parenting (Bögels & Restifo, 2014) and from book on mindfulness daily practice (Bays, 2011) |
|  |  | Mindful observation of child | Exercise from Mindful Parenting (Bögels & Restifo, 2014) |
| 3-4 | Enhancing body awareness | Body scan | Exercise from Mindful Parenting (Bögels & Restifo, 2014) |
|  |  | Promoting bodily awareness during shower, body massage | Exercise from book on mindfulness daily practice (Bays, 2011) |
| 5-6 | Enhancing full body awareness of comfortable and uncomfortable sensations | Sitting meditation | Exercise adapted from Mindful Parenting (Bögels & Restifo, 2014) |
|  |  | Promoting awareness and acceptance of the full range of sensations in parent and child | Exercise from book on mindfulness training (Bays, 2011) |
| 7-8 | Cultivating nonjudgmental  acceptance of emotions, thoughts, and sensations, cultivating compassion | Sitting meditation | Exercise from Mindful Parenting (Bögels & Restifo, 2014) |
|  |  | Loving kindness meditation, cultivating loving kindness for oneself | Exercise adapted from Mindful Parenting (Bögels & Restifo, 2014) |
| 9-10 | Cultivating mindful awareness during stressful interactions  with child | Cultivating mindful awareness via visualization exercise of stressful parent-child interaction, extending | Exercise from Mindful Parenting (Bögels & Restifo, 2014) |
|  |  | Mindful awareness to daily interaction with child, cultivating loving kindness meditation for parent and child | Exercise adapted from Mindful Parenting (Bögels & Restifo, 2014) |
| 11-12 | Cultivating compassionate acceptance of difficult emotions | Cultivating mindful compassion during stressful parent-child interaction, extending mindful compassion during daily stress | Exercise from Mindful Parenting (Bögels & Restifo, 2014) |
|  |  | Interaction with child, cultivating loving kindness meditation for parent and child | Exercise adapted from Mindful Parenting (Bögels & Restifo, 2014) |
| 13-14 | Cultivating compassionate acceptance towards parent and child’s needs, repairing ruptures | Applying mindful compassion during stressful parent-child interaction, identifying needs | Exercise adapted from nonviolence communication (Rosenberg & Gandhi, 1999) |
|  |  | Behind emotions and repairing ruptures, extending mindful compassion acceptance to the child | Exercise from Mindful Parenting (Bögels & Restifo, 2014) |

.

Table 2. Self-reported days of recordings completed by participants

| Audios Completed | No. of Participants in Study 1, N=66 (Percentage) | No. of Participants in Study 2, N=57 (Percentage) |
| --- | --- | --- |
| 1-2 | 3 (4.5%) | 3(5.4%) |
| 3-4 | 1(1.5%) | 10(17.6%) |
| 5-6 | 3(4.5%) | 2(3.5%) |
| 7-8 | 5(7.6%) | 5(8.8%) |
| 9-10 | 5 (7.6%) | 3 (5.4%) |
| 11-12 | 9(13.6%) | 8(14.1%) |
| 13-14 | 40(60.6%) | 26(47.6%) |

Table 3. Participants’ self-reported mindfulness practice at follow-up

| Practice Per Week | No. of Participants in Study 1, N=82 (Percentage) | No. of Participants in Study 2, N=62 (Percentage) |
| --- | --- | --- |
| 0 | 31(37.8%) | 17(27.42%) |
| 1 | 12(14.6%) | 20(32.26%) |
| 2-3 | 21(25.6%) | 18(29.03%) |
| 4-6 | 12(14.6%) | 5(8.06%) |
| 7-9 | 3(3.7%) | 1(1.61%) |
| >10 | 3(3.7%) | 1(1.61%) |

Table 4. Response to the extent participants find the program a difficult one to complete

|  | No. of Participants in Study 1, N=66 (Percentage) | No. of Participants in Study 2, N=57 (Percentage) |
| --- | --- | --- |
| Very Easy | 3 (4.5%) | 2 (3.4%) |
| Easy | 24 (36.4%) | 19 (33.3%) |
| No Comment | 14 (21.2%) | 11 (19.3%) |
| Difficult | 22 (33.3%) | 21 (36.8%) |
| Very Difficult | 3 (4.5%) | 4 (7%) |

Table 5. Reasons for finding the program a difficult one to complete

|  | No. of Participants in Study 1, N=25 (Percentage) | No. of Participants in Study 2, N=24 (Percentage) |
| --- | --- | --- |
| Lack of Time | 18 (72%) | 14 (58.3%) |
| Lack of Undisturbed Space | 3 (12%) | 4 (16.7%) |
| Lack of Motivation | 2 (8%) | 4 (16.7%) |
| Difficult to Concentrate | 1 (4%) | 1 (4.2%) |
| Technical Difficulties | 1 (4%) | 1 (4.2%) |

Table 6. Participants’ General Comments

|  | No. of Participants in Study 1, N=53 (Percentage) | No. of Participants in Study 2, N=47 (Percentage) |
| --- | --- | --- |
| **Overall Positive Comments** | 41((79.6%) | 34 (72.3%) |
| Praised without a specific reason | 28 (53.1%) | 18 (37.5%) |
| Relaxing | 7(14.3%) | 11(22.9%) |
| Appreciated the self-directed element | 1(2%) | 1 (2.1%) |
| Improved emotional regulation | 3(6.1%) | 2 (4.2%) |
| Task is easy to follow | 2 (4.1%) | 1 (2.1%) |
| High coherence of program design | 0(0%) | 1 (2.1%) |
| **Program Difficulty** | 5(10.2%) | 5(10.5%) |
| Hard to find time to complete the program | 2 (4.1%) | 3 (6.3%) |
| Difficult concentrating | 2 (4.1%) | 1 (2.1%) |
| Easy to fall asleep | 1 (2%) | 0(0%) |
| No specific reason was stated | 0(0%) | 1 (2.1%) |
| **Suggested Improvements** | 7 (14.2%) | 8 (16.8%) |
| Audio/Program too short | 3(6.1%) | 3 (6.3%) |
| Instruction speed is too fast/slow | 3(6.1%) | 1(2.1%) |
| Create parent-child version | 1(2%) | 0(0%) |
| Time to receive intervention content (preferably in the morning) | 0(0%) | 1(2.1%) |
| Increase engagement | 0(0%) | 2 (4.2%) |
| Add background music | 0(0%) | 1(2.1%) |
